# Bee Venom and Its Major Component Melittin Attenuated *Cutibacterium acnes*- and IGF-1-Induced Acne Vulgaris via Inactivation of Akt/mTOR/SREBP Signaling Pathway

**DOI:** 10.3390/ijms23063152

**Published:** 2022-03-15

**Authors:** Hyemin Gu, Hyun-Jin An, Mi-Gyeong Gwon, Seongjae Bae, Jaechan Leem, Sun-Jae Lee, Sang-Mi Han, Christos C. Zouboulis, Kwan-Kyu Park

**Affiliations:** 1 Department of Pathology, School of Medicine, Catholic University of Daegu, Gyeongsan 42472, Korea; guhm1207@cu.ac.kr (H.G.); ahj119@cu.ac.kr (H.-J.A.); daldy88@cu.ac.kr (M.-G.G.); zz22@cu.ac.kr (S.B.); pathosjlee@cu.ac.kr (S.-J.L.); 2 Department of Immunology, School of Medicine, Catholic University of Daegu, Gyeongsan 42472, Korea; jcim@cu.ac.kr; 3 Department of Agricultural Biology, National Academy of Agricultural Science, RDA, Wanju 54875, Korea; sangmih@korea.kr; 4 Departments of Dermatology, Venereology, Allergology and Immunology, Dessau Medical Center, Brandenburg Medical School Theodor Fontane, Faculty of Health Sciences Brandenburg, Auenweg 38, 06847 Dessau, Germany; christos.zouboulis@mhb-fontane.de

**Keywords:** bee venom, melittin, *C. acnes*, IGF-1, lipogenesis

## Abstract

Acne vulgaris is the most common disease of the pilosebaceous unit. The pathogenesis of this disease is complex, involving increased sebum production and perifollicular inflammation. Understanding the factors that regulate sebum production is important in identifying novel therapeutic targets for the treatment of acne. Bee Venom (BV) and melittin have multiple effects including antibacterial, antiviral, and anti-inflammatory activities in various cell types. However, the anti-lipogenic mechanisms of BV and melittin have not been elucidated. We investigated the effects of BV and melittin in models of Insulin-like growth factor-1 (IGF-1) or *Cutibacterium acnes* (*C. acnes*)-induced lipogenic skin disease. *C. acnes* or IGF-1 increased the expression of sterol regulatory element-binding protein-1 (SREBP-1) and proliferator-activated receptor gamma (PPAR-γ), transcription factors that regulate numerous genes involved in lipid biosynthesis through the protein kinase B (Akt)/mammalian target of rapamycin (mTOR)/SREBP signaling pathway. In this study using a *C. acnes* or IGF-1 stimulated lipogenic disease model, BV and melittin inhibited the increased expression of lipogenic and pro-inflammatory factor through the blockade of the Akt/mTOR/SREBP signaling pathway. This study suggests for the first time that BV and melittin could be developed as potential natural anti-acne agents with anti-lipogenesis, anti-inflammatory, and anti-*C. acnes* activity.

## 1. Introduction

Acne vulgaris is one of the most common dermatological diseases, affecting nearly 90% of adolescents worldwide. Although acne vulgaris is not life-threatening, it can persist throughout life and leave permanent scarring on the face if not properly treated, thereby causing significant physical and psychosocial morbidities [1,2]. The pathogenesis of acne vulgaris is considered to involve several factors, including inflammation induced by *Cutibacterium acnes* (*C. acnes*) and *Staphylococcus aureus*, follicular hyperkeratinization, and excessive sebum production [3,4]. Excessive sebum production acts as a nutrient for *C. acnes* and plays an important role in the formation of primary lesions related to acne, along with hyperkeratinization of the pilosebaceous duct, hyperproliferation of *C. acnes*, and formation of perifollicular inflammation [5,6].

*C. acnes*, a Gram-positive anaerobic bacterium, is ubiquitous in human skin and is known to play a key role in skin diseases of the pilosebaceous unit characterized by abnormalities of skin microflora [7,8]. Pilosebaceous follicles consist of the hair follicle, hair shaft, and sebaceous gland. The main function of the sebaceous gland and the sebocytes within is to produce and excrete sebum, which contains triglycerides, cholesterol, and free fatty acids [5,9]. Sebaceous gland cell proliferation and sebum production are regulated by a complex system of hormones, as well as other factors such as genetics, environmental and metabolic conditions, stress, diet, and injury [9].

Insulin-like growth factor-1 (IGF-1) is a key hormonal stimulus for the onset of sebum production, enhanced lipogenesis and expression of sterol regulatory element-binding protein-1 (SREBP-1) and proliferator-activated receptor gamma (PPAR-γ) via the phosphatidylinositol 3 kinase (PI3K)/protein kinase B (Akt)/mammalian target of rapamycin (mTOR) signaling pathway in sebocytes [9,10,11]. The mTOR pathway is a central regulator of cellular protein and lipid metabolism, and its key regulatory mTORc1 complex is upregulated in acne sebaceous glands [11,12]. SREBP-1 and PPAR-γ are key transcription factors that regulate the expression of lipogenic genes, such as acetyl coenzyme A carboxylase (ACC), fatty acid synthase (FAS), stearoyl-CoA desaturase 1 (SCD-1), and p70S6k [13,14,15].

Sebaceous gland cells (sebocytes) can produce various cytokines and lipid-derived inflammatory mediators [16] and are believed to participate in the acne inflammatory process [7]. In addition, IGF-1 exposure activated the c-Jun N-terminal kinase (JNK)/p38 signaling pathway in human sebocytes [17]. The JNK and p38 mitogen-activated protein kinase (MAPK) signaling pathways play an important role in basic cellular processes including proliferation, differentiation, survival, and migration [18,19]. Therefore, pharmacologically inhibiting the these signaling pathway could be a desirable approach for affecting sebaceous gland activity and thus inhibiting an important factor in acne pathogenesis.

Currently, medications are moderately effective in the treatment of acne, but they could potentially be associated with serious side effects [20,21]. For example, isotretinoin, one of the most effective treatments, has potentially serious side effects including teratogenicity, dyslipidemia, and liver enzyme abnormalities. Therefore, the need to investigate new antiacne agents has been growing [22], and many researchers are attempting to develop treatments of acne with no side effects and high antibacterial properties, and a systematic and safe agent is needed [23,24].

Bee venom (BV) contains various peptides, including melittin, apamin, adolapin and mast cell degranulating peptide along with enzymes, biological amines, and non-peptide components [25]. Purified BV is a mixture of natural toxins produced by honeybees (*Apis mellifera* L.) [26]. The major component of BV is melittin, which comprises approximately 50% of the dry weight of BV [24]. BV and melittin have been widely used as traditional medicines for various diseases, including arthritis, rheumatism, pain, cancerous tumors, and skin diseases [27,28]. In addition, several studies have examined the biological and pharmacological activities of bee venom and melittin, confirming that they possess radioprotective, anti-inflammatory, antibacterial, antiviral, anticancer and immuno-modulatory activities [23,29].

Our previous study investigated the effects of melittin on *C. acnes*-induced inflammatory responses in vitro and in vivo [23,30]. However, there is not enough evidence regarding the pharmacological mechanism of anti-lipogenesis of BV and melittin in the treatment of acne, and its potential efficacy also needs to be clarified. This study investigated the potential pharmacological effects of BV and its major component, melittin, on models of acne induced by *C. acnes* in vivo and by IGF-1 in human sebaceous gland cells.

## 2. Results

### 2.1. BV and Melittin Alleviate C. acnes-Mediated Acne Lesion Symptoms

**Figure 1:**
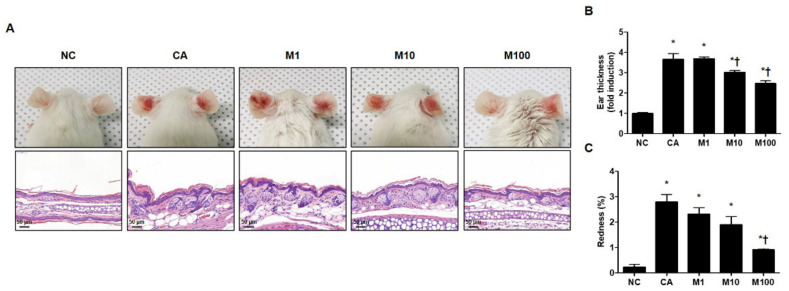
Effect of melittin in *C. acnes*-induced acne models. (**A**) Live *C. acnes* (1 × 10^7^ CFU) were inoculated into the ears of mice together with or without melittin. At 24 h after injection, the representative images of the ear were photographed. (**B**) The mice exhibited cutaneous erythema (or edema and redness), a typical symptom of ear inflammation. Formalin-fixed ear tissue sections were stained with H&E, and the infected areas were quantified. (**C**) Relative evaluation of redness, a typical symptom of acne, was performed. Histological examinations were performed at 400× magnification. Scale bar = 50 μm. The figures are representative of each study group (six mice per group). The results are expressed as means ± SEM. * *p* < 0.05 compared with the NC group. † *p* < 0.05 compared with the CA group. NC: normal control; CA: *C. acnes*; M1, M10, and M100: 1, 10, and 100 μg of melittin mixed with Vaseline and applied topically to the ear surface.

We evaluated the pathophysiological effect of BV or melittin in a *C. acnes*-induced acne mouse model. For this experiment, the mice were injected intradermally with 1 × 10^7^ CFU *C. acnes* and then BV or melittin was applied topically to the ear surface. The ear skin was excised after 24 h. H&E staining was performed to evaluate morphological changes in the sebaceous glands. The mice exhibited cutaneous erythema, a typical symptom of acne (Figure 1A and Figure S1A). However, the ears treated with BV and melittin showed markedly reduced edema and redness compared with ears injected with only *C. acnes* (Figure 1B,C and Figure S1B,C).

### 2.2. BV and Melittin Attenuate the C. acnes-Induced Expression of SREBP and PPAR-γ

**Figure 2:**
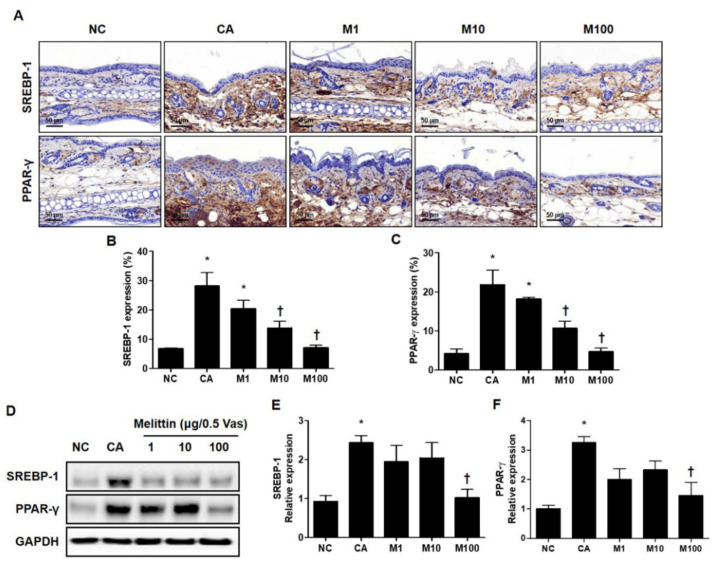
Regulation of SREBP-1 and PPAR-γ in *C. acnes*-induced acne model by melittin. The representative IHC analysis images showed that the expression of lipogenesis markers SREBP-1 and PPAR-γ was inhibited by the administration of melittin in a *C. acnes*-induced acne model. (**A**) The graphs indicate the relative percentage of the expression of SREBP-1 and PPAR-γ in the ear skin sections. The integrated optical densities were measured from at least five random fields per section. (**B**,**C**) The integrated optical densities were measured from at least five random fields per section. (**D**) The Western blot analysis shows the protein expressions of SREBP-1, PPAR-γ, and GAPDH in the ear skin of mice in each group. GAPDH was used to confirm equal sample loading. The bar graph shows the quantitative signal intensity of (**E**) SREBP-1 and (**F**) PPAR-γ after normalization with GAPDH. Histological examinations were performed at 400× magnification under light microscopy. The results are expressed as means ± SEM. * *p* < 0.05 compared with the NC group. † *p* < 0.05 compared with the CA group. NC: normal control; CA: *C. acnes*; M1, M10, and M100: 1, 10, and 100 μg of melittin mixed with Vaseline.

To determine whether BV or melittin inhibited the expression of these lipogenic regulators, the expression of PPAR-γ and SREBF-1 was confirmed by immunohistochemical (IHC) and Western blot analyses (Figure 2A and Figure S2A). Using the results of the IHC analysis, the *C. acnes*–induced acne model significantly increased the expression of lipogenic regulators, such as PPAR-γ and SREBP-1, whereas treatment with BV or melittin attenuated these expressions in a dose-dependent manner (Figure 2B and Figure S2B). Similarly, BV treatment reduced the expression of SREBP-1 in a concentration-dependent manner, and it was confirmed that the expression of PPAR-γ was significantly reduced in BV100 μg (Figure S2B,C).

### 2.3. BV and Melittin Prevent the Activation of Lipogenesis-Related Genes in C. acne-Induced Mouse Model

**Figure 3:**
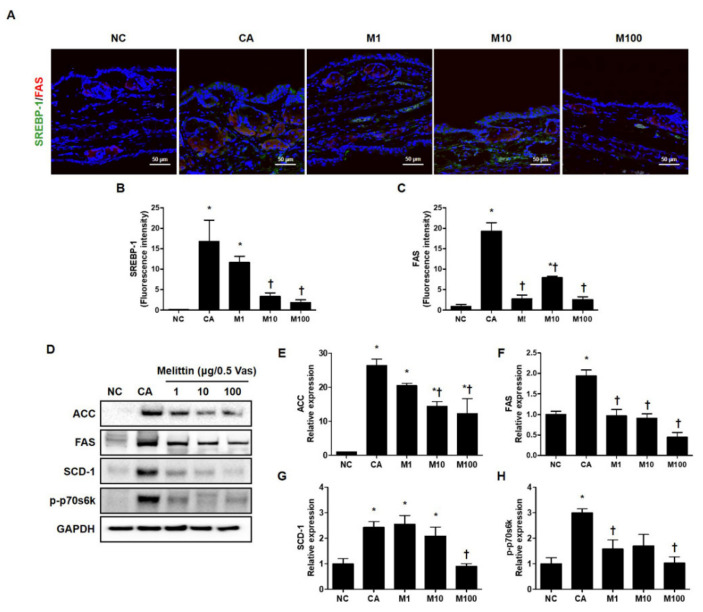
Effect of melittin in *C. acnes*-mediated expression of the protein encoded by the lipogenesis-related gene. The inhibitory effect of melittin on lipid synthesis in *C. acnes*-induced acne mice was examined by immunofluorescence. (**A**–**C**) The representative immunofluorescence images show the effect of melittin on the *C. acnes*-induced activation of SREBP-1 (labeled with Alexa Fluor 488, green) and FAS (labeled with Alexa Fluor 555, red). The nuclei were labeled with 4,6-diamidino-2-phenylindole dihydrochloride (DAPI) (blue); Scale bar = 50 μm. (**D**) The protein level for lipid-associated factor was evaluated by the Western blot analysis. The blots were incubated with antibodies specific to ACC, FAS, SCD-1, and p70s6k. GAPDH was used as a loading control. (**E**–**H**) We next quantified the protein levels. The results are expressed as means ± SEM. * *p* < 0.05 compared with the NC group. † *p* < 0.05 compared with the CA group. NC: normal control; CA: *C. acnes*; M1, M10, and M100: 1, 10, and 100 μg of melittin mixed with Vaseline.

To verify the attenuation in *C. acnes*-induced lipogenesis by treatment with BV or melittin, we next evaluated the lipid synthesis-related genes, such as SREBP-1 and FAS, using immunofluorescence (Figure 3A–C and Figure S3A–C). In addition, we evaluated the protein encoded by lipogenesis-related genes such as ACC, FAS, and SCD-1 and phosphorylated forms of p70s6k using western blot analysis (Figure 3D and Figure S3D). Our results show that treatment with BV or melittin markedly reduced SREBP and FAS, indicating that BV and melittin inhibited the expression of lipogenic regulators and lipid accumulation in vivo (Figure 3D–H and Figure S3D). Our findings suggest that BV and melittin can reduce the expression of lipogenic regulators and lipid production response induced by *C. acnes*. To identify downstream regulators responsible for the BV- or melittin-mediated suppression of the SREBP pathway, metabolic factors involved in lipogenesis, such as FAS, SCD-1, and ACC were detected using the Western blot analysis. As expected, the protein expressions of ACC, FAS, SCD-1, and p-p70s6k downstream of SREBP-1 were significantly increased in the *C. acnes* group, whereas they were suppressed in the BV or melittin group.

### 2.4. Melittin Decreases Activation of C. acnes-Induced Lipogenesis Pathway

To elucidate the underlying mechanism by which melittin attenuates *C. acnes*-induced lipogenesis, we evaluated the lipid synthesis-related signaling cascades. Using the results of the Western blot analysis, we confirmed that the PI3K/mTOR signaling pathway, an important signaling pathway contributing to lipid synthesis induced by *C. acnes*, was significantly increased through the mouse model. In contrast, treatment with melittin effectively attenuated *C. acnes*-mediated lipid production response in the mouse model through the inhibition of the PI3K/mTOR pathway. As shown in Figure S4A, it was confirmed that the activity of the factors involved in the lipid synthesis pathway, such as PI3K and mTOR was significantly reduced at the 100 μg of melittin. As a result, melittin inhibited the *C. acnes*-induced lipogenesis pathway by suppressing the expression of p-PI3K and p-mTOR. Collectively, these results suggest that melittin specifically ameliorates *C. acnes*-induced lipogenesis in the mouse model.

### 2.5. BV and Melittin Suppress C. acnes-Mediated Pro-Inflammatory Cytokines

The ear tissue was harvested to further investigate the inflammatory response of the skin. Subsequently, to investigate the anti-inflammatory effects of BV and melittin in acne, we first analyzed the expression of inflammatory cytokines that could be detected by Western blot analysis. As shown in Figures S4B and S5A, the expression of pro-inflammatory cytokines, such as TNF-α, IL-1β, IFN-γ, and IL-6 was significantly upregulated in the *C. acnes*-treated group compared to the normal control group. In contrast, treatment with BV or melittin effectively decreased the levels of pro-inflammatory cytokines, such as TNF-α, IL-1β, IFN-γ, and IL-6 in vivo (Figures S4B–F and S5A–D). We further analyzed the serum levels of IFN-γ by ELISA. As shown in Figure S4G, melittin suppressed the expression of IFN-γ in mice serum.

### 2.6. Effects of BV and Melittin on Cell Viability

CCK-8 assay was conducted to determine the cytotoxicity of BV and melittin at different doses. The cells were treated with BV or melittin at different concentrations and assessed at different time points. The immortalized human facial sebaceous gland cell line SZ95 cells were relatively stable, with no significant change in viability up to 16 h.

**Figure 4:**
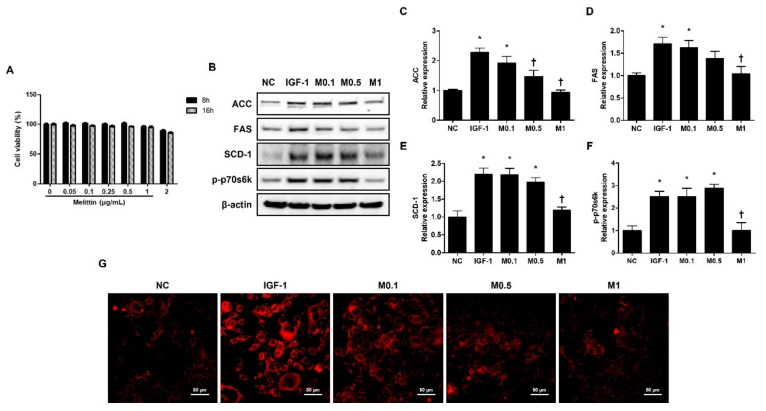
Melittin inhibits IGF-1-induced lipogenic factors in SZ95 cells. (**A**) Cytotoxic effects of melittin on SZ95 cells. Cell viability was determined by CCK-8 assay (*n* = 5). (**B**) The effects of melittin on the expressions of lipogenic factors, such as ACC, FAS, SCD-1, and p-p70s6k in SZ95 cells that were stimulated by IGF-1. (**C**–**F**) We quantified the protein levels next. β-actin was presented as loading control. Intracellular lipids were detected by Nile red staining. (**G**) Representative Nile Red stain images were detected in intracellular lipids at 400× magnification. Scale bar = 50 μm. The results are expressed as means ± SEM of three independent determinations. * *p* < 0.05 compared with the NC group. NC group. † *p* < 0.05 compared with the IGF-1 group. NC: normal control; IGF-1: insulin-like growth factor-1; M0.1, M0.5, and M1: 0.1, 0.5, and 1 μg·mL^−^^1^.

However, the cells treated with 200 ng·mL^−1^ BV or 2 μg·mL^−1^ melittin showed a slight decrease in viability at 16 h compared to that without treatment (Figure 4A and Figure S6A). In accordance with this result, treatment with BV or melittin for the SZ95 cells was performed for 16 h.

### 2.7. BV and Melittin Suppress IGF-1-Induced Lipid Synthesis in Human Sebocytes

**Figure 5:**
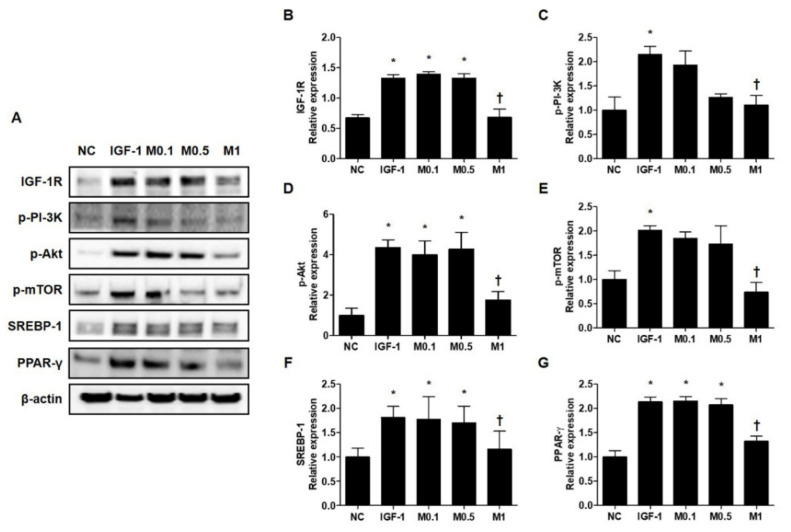
Melittin effectively inhibits the IGF-1R/Akt/mTOR/SREBP signaling pathway in IGF-1-treated SZ95 cells. Western blot analysis shows that the phosphorylation of p-IGF-1R, p-PI3K, p-Akt, and p-mTOR is suppressed by treatment with melittin. (**A**) Representative western blot analysis images show that treatment with melittin almost completely blocked the activation of IGF-1R/Akt/mTOR/SREBP signaling pathway after treatment of SZ95 cells with IGF-1. β-actin was presented as a loading control. (**B**–**G**) We quantified the protein levels next. The results are expressed as means ± SEM. * *p* < 0.05 compared with the NC group. † *p* < 0.05 compared with the IGF-1 group. NC: normal control; IGF-1: insulin-like growth factor-1; M0.1, M0.5, and M1: 0.1, 0.5, and 1 μg·mL^−^^1^.

The SZ95 exhibits the properties of normal sebocytes, such as the expression of characteristic sebaceous gland proteins and the development of Nile Red stain-positive lipid droplets in their cytoplasm [31]. To investigate the effect of BV and melittin on lipid synthesis in human sebocytes, human sebocytes, SZ95 cells, were cultured with IGF-1, and they showed increased levels of ACC, FAS, SCD-1, and p-p70s6k. In addition, the upregulation of ACC, FAS, SCD-1, and p-p70s6k treatment with IGF-1 was reversed by BV and melittin (Figure 4B and Figure S6B). Both BV and melittin suppressed the expression of FAS, ACC, SCD-1, and p-p70s6k at 100 ng·mL^−^^1^ and 1 μg·mL^−^^1^ concentrations, respectively (Figure 5C–F and Figure S6C–F). These results suggest that BV or melittin may suppress lipogenesis in human sebocytes. To examine the effect of BV and melittin in greater detail, we investigated lipid accumulation using Nile Red staining in the SZ95 cells. Upon investigation, it was found that the group treated with IGF-1 had significantly higher lipid accumulation compared to the group treated with BV or melittin (Figure 4G and Figure S6G).

### 2.8. IGF-1 Induces Lipid Synthesis via the Activation of IGF-1R/Akt/mTOR/SREBP Signaling Pathway, and This Effect is Suppressed by BV and Melittin

These results prompted us to examine the molecular mechanism by which BV and melittin suppress lipid synthesis. We tested the hypothesis of whether or not the IGF-1R/PI3K/Akt/SREBP pathway, a major pathway responsible for regulating of lipid metabolism, was involved in the anti-lipogenic effects of BV and melittin. First, we examined whether or not BV and melittin inhibit the activation of IGF-1R under the stimulation of IGF-1 in SZ95 sebocytes. We found that IGF-1 increased the levels of phosphorylated IGF-1R, and treatment with BV or melittin markedly decreased the levels of the phospho-IGF-1R protein (Figure 5A and Figure S7A). To provide additional evidence that BV and melittin target IGF-1R signaling, we next examined if BV and melittin inhibit well-known downstream effector pathways of IGF-1R. The levels of phosphorylated Akt were, as expected, increased by IGF-1. There was a remarkable change in the level of phospho-Akt following treatment with BV or melittin. We further found that phospho-mTOR, SREBP, and PPAR-γ critical downstream elements in the Akt pathway were regulated by BV and melittin (Figure 5B–G and Figure S7B–G). Together, our data indicate that BV and melittin suppressed sebum through inhibition of the IGF-1R/PI3K/Akt/SREBP-1 signaling pathway in SZ95 sebocytes offering therapeutic strategies to target lipogenesis in acne.

### 2.9. BV and Melittin Regulate IGF-1-Induced Inflammatory Cytokine Production in SZ95 Cells

**Figure 6:**
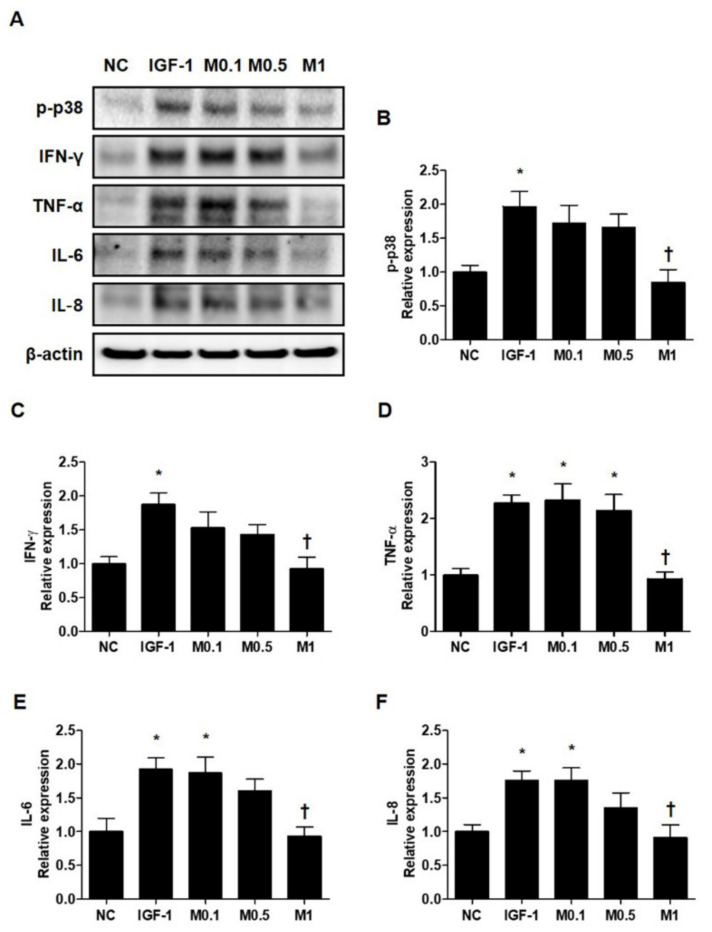
Melittin effectively inhibits the pro-inflammatory cytokine in IGF-1-treated SZ95 cells. (**A**) Representative cropped Western blot analysis images show that melittin inhibited the IGF-1-induced protein expressions of p38 MAPK and pro-inflammatory cytokines, such as TNF-α, IL-1β, IL-8, and IFN-γ. β-actin was presented as a loading control. (**B**–**F**) We quantified the protein levels next. The results are expressed as means ± SEM. * *p* < 0.05 compared with the NC group. † *p* < 0.05 compared with the IGF-1 group. NC: normal control; IGF-1: insulin-like growth factor-1; M0.1, M0.5, and M1: 0.1, 0.5, and 1 μg·mL^−1^.

Pro-inflammatory cytokines, such as IFN-γ, TNF-α, IL-6, and IL-8, are already known to be upregulated in acne lesions [32,33]. To investigate the effects of BV and melittin on SZ95 sebocytes in greater detail, we examined the expression of various pro-inflammatory cytokines. Similar to the results for anti-lipogenesis, 20 ng·mL^−1^ IGF-1 induces several pro-inflammatory cytokines including p-p38, IFN-γ, TNF-α, IL-6, and IL-8 in SZ95 sebocytes (Figure 6A and Figure S8A). In contrast, BV and melttin 100 ng·mL^−1^ and 1 μg·mL^−1^, respectively, suppress the inflammatory response induced by IGF-1 in SZ95 sebocytes, a well-established in vitro model of pro-inflammatory acne (Figure 6B–F and Figure S8B–S8F).

## 3. Discussion

Acne vulgaris is among the most common chronic skin diseases of the pilosebaceous follicle, involving the abnormal keratinization of keratinocytes and proliferation of *C. acnes*, the causative agent of perturbation of sebaceous gland function [34]. Colonization of the follicle with *C. acnes* is another critical pathological factor for acne [5,35,36], which is involved in the inflammatory response and lipogenesis within the pilosebaceous unit [37,38]. Sebaceous glands are widely considered to contribute to the lipid barrier of the skin by producing the lipid-rich sebum [39,40], and within the sebaceous follicle, abnormal viability, proliferation, and differentiation of keratinocytes and sebocytes result in increased sebum production. Alterations in sebum lipid composition play a crucial role in the clinical development and aggravation of acne. Excessive sebum production is attributable to inflammatory disorders associated with the excessive growth of *C. acnes* [41]. Despite the advanced understanding of the pathophysiological mechanisms underlying acne, the development of drugs targeting several pathological processes is limited. Antibiotic therapy, one of the treatment options, is a typical treatment for inflammatory skin diseases caused by *C. acnes* and is provided to suppress inflammation or kill bacteria. However, these antibiotics have been known to induce side effects [26]. Therefore, many researchers have tried to develop therapeutic agents for acne that have no side effects, but high antibacterial activity.

BV and melittin have therapeutic and toxic effects depending on the concentrations. Some studies have shown that BV and melittin are known to cause pain and inflammation [42]. These studies were performed using relatively high concentrations of bee venom and melittin. On the other hand, we have demonstrated that relatively low concentrations (lower than 1/10–1/100 of hypersensitivity dose, maybe 1 bee sting dose) of bee venom and melittin inhibit inflammation in various diseases model [23,24,26]. Our previous study demonstrated the effects of melittin on *C. acnes*-induced inflammatory responses in vitro and in vivo [23]. Recent studies have shown that treatment with BV can induce a significant anti-inflammatory response mediated by the inhibition of inflammatory mediators, which is similar to what is achieved with the administration of non-steroidal anti-inflammatory drugs [23,24].

The activation of SREBP-1 and PPAR-γ is accompanied by an increase in the transcription of several lipogenic genes. The lipogenic genes ACC, FAS, SCD-1, and p70s6k are target genes of SREBP-1 and PPAR-γ, involved in fatty acid biosynthesis [43,44]. Li et al. [45] demonstrated that the protein expression and activity of ACC, FAS, and SCD-1 were suppressed when the aberrant expression of SREBP-1 was suppressed. Furthermore, Romano et al. [46] demonstrated that downregulation of SREBP-1 and PPAR-γ suppressed lipogenesis-specific gene expression. Whether the expression of these major lipogenic factors is regulated is one of the main indicators to determine the inhibitory effect of pharmacologically active substances on lipogenesis [47]. Thus, specific SREBP-1 and PPAR-γ antagonists might be regarded as candidates for anti-acne agents. In this study, the IGF-1-induced expression of SREBP-1 and PPAR-γ in SZ95 sebocytes was diminished by BV and melittin. The *C. acnes*-induced expression of SREBP-1 and PPAR-γ in vivo was diminished by BV and melittin. Likewise, the protein activation of ACC, FAS, SCD-1, and p70s6k was statistically significantly inhibited by BV and melittin following injection with *C. acnes*. We also showed that IGF-1 increased lipid synthesis in SZ95 sebocytes, however, when cells were treated with BV and melittin, IGF-1 failed to induce lipogenesis. Therefore, we confirmed this anti-lipogenic effect by showing that BV and melittin also decreased intracellular lipid accumulation without notable cytotoxicity in vivo and in vitro. Through this, we hypothesized that BV and melittin could be developed as anti-acne agents with anti-lipogenic effects associated with the inactivation of SREBP-1 and PPAR-γ.

To support this hypothesis, it was confirmed that the activation of IGF-1R stimulated by IGF-1 was inhibited by BV and melittin, and it mediated the downregulation of IGF-1R/PI3K/Akt/mTOR signaling activation. Smith et al. [17] demonstrated that the inhibition of the PI3K pathway also blocked the IGF-1-induced transcription of SREBP target genes and sebocyte lipogenesis. These data indicated that IGF-1 transmits its lipogenic signal in sebocytes through the activation of Akt. As such, Smith et al. [17] suggested that specific targeted interruption of this pathway in the sebaceous gland could be a desirable approach to reducing sebum production and improving acne. Yoon et al. [48] demonstrated that the downregulation of IGF-1R/PI3K/Akt/SREBP-1 signaling pathway had sebosuppressive effects mainly in sebocytes. Furthermore, Im et al. [43] have shown that the inactivation of IGF-1R/Akt/mTOR can overcome the stimulatory effects of IGF-1 and may be a suitable anti-IGF-1 agent. Hence, our findings suggest that BV and melittin inactivate IGF-1R/Akt/mTOR/SREBP and this is likely to be important in the anti-lipogenic action against *C. acnes* or IGF-1, offering therapeutic strategies to target lipogenesis in acne. In acne, *C. acnes*–induced inflammatory response the around pilosebaceous gland, mainly through the secretion of various pro-inflammatory cytokines, represents a key pathogenic factor leading to disease initiation and aggravation [5,36,49].

Previous studies have found that *C. acnes* stimulates the production of pro-inflammatory cytokines such as TNF-α, IL-1β, IL-6, and IL-8 [23,30,50]. TNF-α and IL-6 are also potent inflammatory molecules that have endocrine effects in either acute or chronic inflammation [51]. TNF-α is the upstream modulator of mTOR [52]. Although the suppression of sebum production is a principal treatment objective, reducing the levels of pro-inflammatory fatty acids specifically may offer a critical contribution to acne improvement. As these inflammatory mediators are thought to increase the inflammatory state of acne and to aggravate the initial acne lesion, we investigated whether or not BV and melittin could inhibit pro-inflammatory cytokine production in the *C. acnes*-induced acne model and IGF-1-stimulated SZ95 cells. We investigated the effects of BV and melittin on IGF-1-induced inflammatory cytokine production in SZ95 cells. Our findings provide evidence that BV and melittin have a significant suppressive effect on IGF-1-induced expression of TNF-α, IL-1β, IL-6, and IL-8 in SZ95 cells. In addition, our findings provide evidence that both BV and melittin have a marked suppressive effect on the production of *C. acnes*-induced TNF-α, IL-1β, IL-6, and IL-8 in vivo. In agreement with our results, Lee et al. [23] demonstrated that *C. acnes* leads to the degradation of IkB, to stimulation of the MAPK pathway, and to increased pro-inflammatory cytokine production in keratinocytes. The p38 MAPK signaling pathways play an important role in basic cellular processes, including proliferation, differentiation, survival, and migration [18,19]. It has been reported that p38 is a lipogenic signal molecule, and specific p38 inhibitors block pogenesis and FAS expression [53]. Furthermore, p38 mediates lipogenesis in hepatocytes [54]. Similar results to those of this study are also shown by Kwon et al. [9], who demonstrated that the activation of p38 induced lipogenesis and the inactivation of p38 mitigated lipogenesis and sebum production. This finding suggests that BV and melittin, as well as antagonizing IGF-1, may inhibit inflammation by additional mechanisms. Therefore, we suggest that the inactivation of p38 is a candidate agent for the treatment of acne. Together, we suggest that BV and melittin may represent a new therapeutic opportunity in acne vulgaris.

## 4. Materials and Methods

### 4.1. Materials

BV was supplied by Chung Jin Biotech Co. (Ansan, Korea) and melittin was supplied by Enzo Life Sciences (Farmingdale, NY, USA).

### 4.2. Preparation of Bacteria

*C. acnes* (ATCC 6919; American Type Culture Collection, Rockville, MD, USA) were obtained from the Korean Culture Center of Microorganisms (Seoul, Korea) and cultured at 37 °C on Brain Heart Infusion Medium (BD Diagnostics, Sparks, MD, USA) under anaerobic conditions at 37 °C until reaching OD_600_ = 1.0 (logarithmic growth phase). The log phase bacterial culture was centrifuged at 5000× *g* at 4 °C for 15 min. Subsequently, the culture supernatant was removed.

### 4.3. Animal Model

Eight-week-old ICR mice were randomly subdivided into 8 groups (6 mice/group) from Samtako (Osan, Korea) and were individually housed in polycarbonate cages and maintained under constant temperature (22 ± 2 °C) and humidity conditions (55%). The mice were allowed free access to food and water and were maintained in an environment with a 12:12-h light/dark cycle. Normal control (NC): phosphate-buffered saline (PBS) only and *C. acnes* (CA): Living *C. acnes* (1.0 × 10^7^ colony forming unit (CFU)/20 μL in PBS) were intradermally injected into both the left and right ears. BV1, BV10, and BV100: Living *C. acnes* were intradermally injected into both the left and right ears. After injection, BV (1, 10, and 100 μg mixed with 0.05 g Vaseline) was applied to the surface of the ear skin of each group. Mel1, Mel10, and Mel100: Living *C. acnes* were intradermally injected into both the left and right ears. After injection, melittin (1, 10, and 100 μg mixed with 0.05 g Vaseline) was applied to the surface of the ear skin of each group at the end of each treatment period (24 h later), the mice were sacrificed by CO_2_ asphyxiation and the ears were excised.

### 4.4. Cell Culture

The SZ95, immortalized human sebaceous gland cell line, was kindly provided by Dr. Christos C. Zouboulis [31]. The cells were maintained in Sebomed basal medium (Biochrom, Berlin, Germany) supplemented with 5 ng·mL^−1^ recombinant human epidermal growth factor and 10% fetal bovine serum at 37 °C in a humidified incubator (Sanyo, Osaka, Japan) containing 5% CO_2_. The cells were then treated with serum-free culture medium containing 20 ng·mL^−1^ IGF-1 (PeproTech, Rocky Hill, NJ, USA) in the presence or absence of different concentrations of BV (1, 10, and 100 ng·mL^−1^) and memL (0.1, 0.5, and 1 μg·mL^−1^) for 16 h.

### 4.5. Cell Viability Assays

The viability of SZ95 was assessed using Cell Counting Kit (CCK)-8 assays (Dojindo, Kumamoto, Japan). The SZ95 cells were seeded in 96-well culture plates at 5.0 × 10^4^ cells·mL^−1^ and allowed to attach for 24 h. The medium was replaced with serum-free media. Cells were treated with serum-free media containing different concentrations of BV (1, 10 and 100 ng·mL^−1^) and melittin (0.1, 0.5 and 1 μg·mL^−1^) for 8 and 16 h. After experimental treatment, 10 μL of the WST-8 solution (2-[2-methoxy-4-nitrophenyl]-3-[4-nitrophenyl]-5-[2,4-disulfophenyl]-2H-tetrazolium, monosodium salt) was added to each well, and the SZ95 cells were incubated for an additional 2–4 h at 37 °C. The absorbance values were measured at 450 nm using a microplate reader.

### 4.6. Western Blot Analysis

Total protein samples were prepared from the ear skin and the cultured SZ95 using CelLytic M (Sigma-Aldrich, St. Louis, MO, USA) according to the instruction manual. After incubation for 30 min on ice, the total extract was centrifuged at 12,000 rpm at 4 °C for 10 min and the supernatant was collected. The protein concentration of the samples was measured using a Bradford assay (Bio-Rad Laboratories, Hercules, CA, USA) at 595 nm using a spectrophotometer.

Protein samples were separated using precast gradient polyacrylamide gels (Bolt™ 4–12% Bis-Tris Plus Gels; Thermo Fisher Scientific, Waltham, MA, USA) and transferred to the nitrocellulose membrane (GE Healthcare, Chicago, IL, USA). The membrane was blocked in 5% bovine serum albumin (BSA). The membrane was probed with primary antibodies and horseradish peroxidase-conjugated secondary antibody. The signal intensity was measured with an image analyzer (ChemiDoc™ XRS+; Bio-Rad Laboratories) and quantified using Image Lab software (Bio-Rad Laboratories). The protein expression values were normalized to β-actin (Sigma-Aldrich) expression values. The primary antibodies used were as follows: anti-IGF-1R, anti-phospho-Akt, anti-phospho-mTOR, anti-FAS, anti-ACC, anti-phospho-p70s6k, anti-phospho-p38, anti-Glyceraldehyde 3-phosphate dehydrogenase (GAPDH) (1:1000, Cell Signaling Technology, Danvers, MA, USA), anti-phospho-PI3K, anti-SREBP, anti-PPAR-γ, anti-SCD-1, anti-interleukin (IL)-8, anti-IL-1β (1:1000, Santa Cruz Biotechnology, Dallas, TX, USA), anti-tumor necrosis factor (TNF)-α, anti-interferon (IFN)-γ, anti-IL-6 (Abcam, Cambridge, UK), and anti-β-actin (1: 1000, Sigma-Aldrich).

### 4.7. Nile Red Staining

To detect sebaceous lipids, the SZ95 cells were seeded in 6-well culture plates at a density of 5 × 10^5^ cells per well and cultured overnight; following this, they were treated with BV or melittin in the presence or absence of IGF-1 for a total of 16 h. For Nile Red staining, a stock solution of Nile Red (Sigma-Aldrich; 1 mg·mL^−1^ in acetone) was diluted to a final concentration of 10 μg·mL^−1^ in PBS. The cells were fixed in 4% formaldehyde at room temperature for 10 min, stained with Nile Red solution for 15 min at 37 °C, and washed with PBS. The stained cells were viewed under a confocal microscope system (Nikon A1 microscope equipped with a digital camera; Nikon, Tokyo, Japan).

### 4.8. Histological Analysis

The ear skin was fixed in 4% formaldehyde for at least 24 h at room temperature. It was dissected, dehydrated, and embedded in paraffin. Thereafter, thin (4 μm) sections were mounted on glass slides and stained with hematoxylin and eosin (H&E). All the slides were examined under a Pannoramic^®^ MIDI slide scanner (3DHISTECH, Budapest, Hungary).

### 4.9. Immunohistochemical Analysis

The paraffin-embedded tissue sections on the slides were deparaffinized. They were then incubated with a primary antibody for 1 h at 37 °C. The primary antibodies were anti-SREBP-1 (1:2000, Abcam) and anti-PPAR-γ (1:800, Cell singling). The signal was visualized using the EnVision System (DAKO, Santa Clara, CA, USA) for 30 min at 37 °C; 3,3′-diaminobenzidine tetrahydrochloride was used as the coloring reagent, and hematoxylin was used as the counterstain. The slides were examined with a Pannoramic^®^ MIDI slide scanner, and the integrated optical density was analyzed using i-Solution DT software (IMT i-Solution Inc., Daejeon, Korea).

### 4.10. Immunofluorescence Analysis

The paraffin-embedded skin tissue sections were deparaffinized with xylene and dehydrated in gradually decreasing concentrations of ethanol. The tissue sections were then placed in a blocking serum (5% BSA in PBS) at room temperature for 1 h. A primary antibody was incubated at room temperature for 2 h, and a secondary antibody incubation was performed at room temperature for 1 h. The antibodies included anti-SREBP-1 (1:200, Santa Cruz Biotechnology), anti-FAS (1:100, Cell Signaling Technology), the secondary antibody conjugated with Alexa Fluor 488 (1:200, Thermo Fisher Scientific), and the secondary antibody conjugated with Alexa Fluor 555 (1:200, Thermo Fisher Scientific). The sections were then counterstained with Hoechst 33342. The slides were mounted using the VECTASHIELD Mounting Medium (VECTOR Laboratories, Burlingame, CA, USA). The stained slides were viewed under a confocal microscope system (Nikon A1 microscope equipped with a digital camera).

### 4.11. Enzyme-Linked Immunosorbent Assay

The blood collected by cardiac puncture was allowed to clot for 1 h at room temperature. The clots were removed by centrifugation (4000 rpm, 20 min). Sera were obtained from the supernatants after centrifugation for Enzyme-linked immunosorbent assay (ELISA). The levels of IFN-γ (R&D systems, Minneapolis, MN, USA) in mouse serum samples were measured using ELISA kits according to the manufacturer’s instructions. The absorbance at 450 nm was measured with an ELISA reader (BMG Labtech, Ortenaukreis, Germany).

### 4.12. Data and Statistical Analysis

The data and statistical analysis in this study comply with the recommendations on experimental design and analysis in pharmacology. All data are presented as means ± standard error of the mean (SEM). Statistical significance was tested using Prism 5 (GraphPad Software Inc., San Diego, CA, USA). Group means were compared by one-way ANOVA with Tukey’s multiple comparison test. Tukey’s tests were run only when F achieved *p* < 0.05 and there was no significant variance inhomogeneity. Differences with *p* < 0.05 were considered significant.

## 5. Conclusions

We investigated the effects of BV and melittin on a *C. acnes*- or IGF-1-induced lipogenesis and inflammatory reaction in vivo and in vitro. Our findings imply that BV and melittin disturb the Akt/mTOR/SREBP signaling pathway in performing an anti-lipogenesis and anti-inflammatory function because the Akt/mTOR/SREBP signaling pathway controls protein synthesis through the activation and phosphorylation of ACC, FAS, SCD-1, and p70s6k. Through this, we have demonstrated that exposure to *C. acnes* or IGF-1 induced lipogenesis, sebum production, and inflammatory reaction via the activation of Akt/mTOR/SREBP signaling in vivo and in vitro, and this effect was suppressed by BV and melittin. Hence, BV and melittin effectively suppressed inflammatory responses by regulating the Akt/mTOR/SREBP signaling and suppressed lipogenesis by modulating lipogenesis. Thus, BV and melittin might be used as a potential anti-acne agent targeting inflammation and lipogenesis triggered.

## Data Availability

Not applicable.

## Supplementary Materials

The following are available online at https://www.mdpi.com/article/10.3390/ijms23063152/s1.
