# Supplementary material for: Bee Venom and Its Major Component Melittin Attenuated Cutibacterium acnes- and IGF-1-Induced Acne Vulgaris via Inactivation of Akt/mTOR/SREBP Signaling Pathway
Source: Int J Mol Sci. 2022 Mar 15;23(6):3152. doi: 10.3390/ijms23063152 (PMC8953527; doi:10.3390/ijms23063152)
Supplement: Supplementary file 1 [file ijms-23-03152-s001.zip › ijms-1609468-supplementary.pdf]

Bee venom and its major component melittin attenuated *Cutibacterium acnes*- and IGF-1-induced acne vulgaris via inactivation of Akt/mTOR/SREBP signaling pathway

Hyemin Gu[1], Hyun-Jin An[1], Mi-Gyeong Gwon [1], Seongjae Bae [1], Jaechan Leem [2], Sun-Jae Lee [1], Sang Mi Han[3], Christos C. Zouboulis[4] and Kwan-Kyu Park [1,*]

* Correspondence: Kwan-Kyu Park; Department of Pathology, School of Medicine, Catholic University of Daegu, 42472, Republic of Korea; Tel.: +82-53-650-4149; E-mail: kkpark@cu.ac.kr

Supplementary figure legends

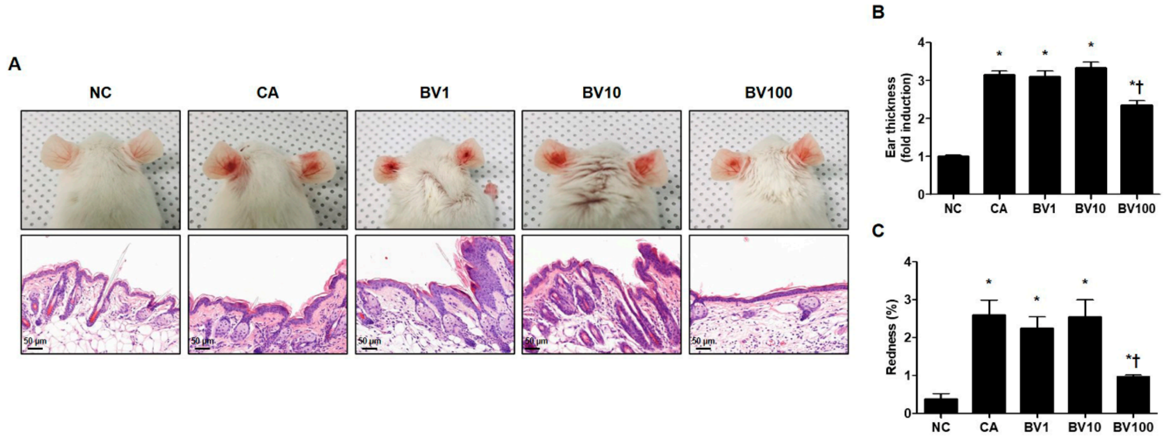

Figure S1. Effect of BV in *C. acne*-induced acne models

Live *C. acnes* (1 × 10^7 CFU) were inoculated into the ears of mice together with or without BV. At 24 h after injection, the representative images of the ear were photographed. (**A**) The mice exhibited cutaneous erythema, a typical symptom of ear inflammation. (**B**) Formalin-fixed ear tissue sections were stained with H&E, and the infected areas were quantified. (**C**) Relative evaluation of redness, a typical symptom of acne, was performed. Histological examinations were performed at 400× magnification. Scale bar = 50 μm. The figures are representative of each study group (six mice per group). The results are expressed as means ± SEM. *$p < 0.05$ compared with the NC group. NC: normal control; CA: C. acnes; BV1, BV10, and BV100: 1, 10, and 100 μg of bee venom mixed with Vaseline.

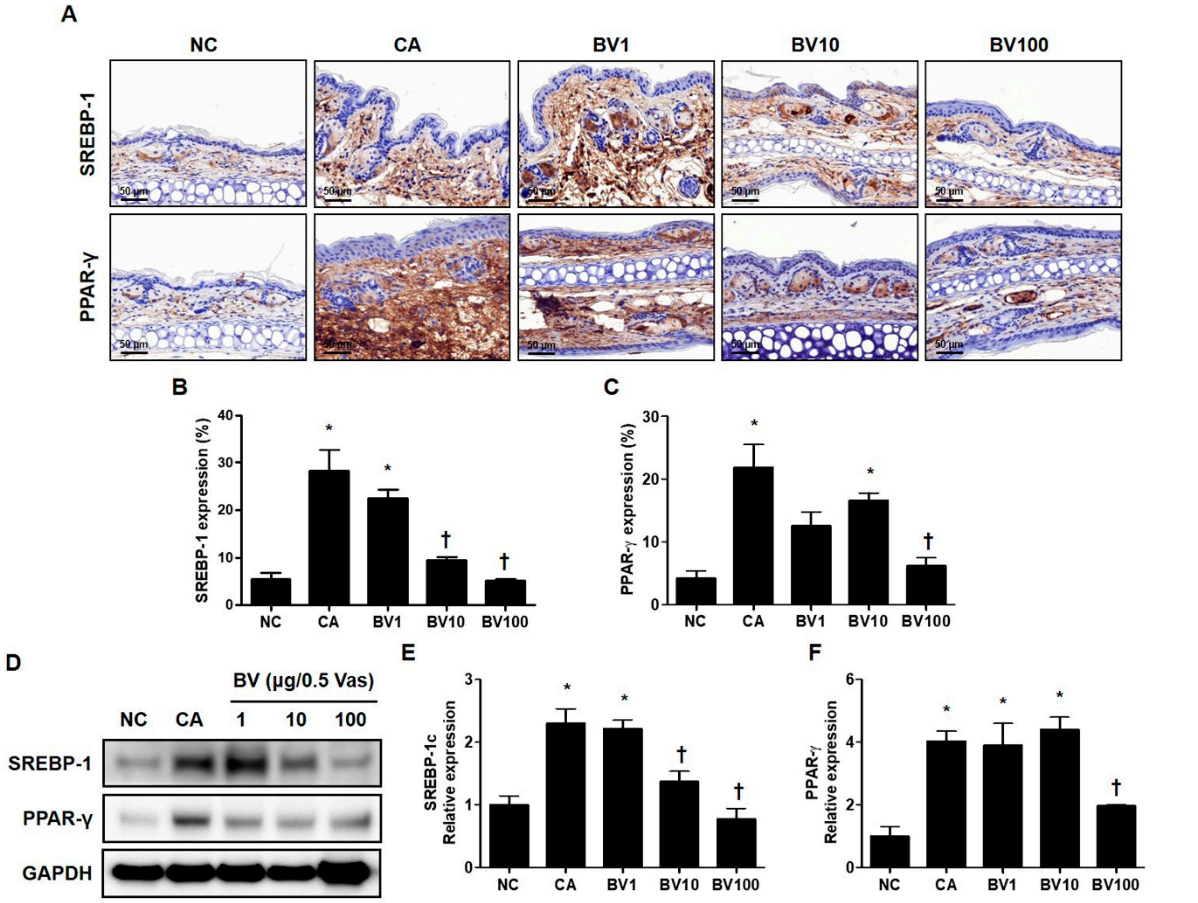

Figure S2. Regulation of SREBP-1 and PPAR-γ in *C. acnes*-induced acne model by BV

The representative IHC analysis images showed that the expression of lipogenesis markers SREBP-1 and PPAR-γ was inhibited by the administration of BV in *C. acnes*-induced acne model. The graphs indicate the relative percentage of the expression of SREBP-1 and PPAR-γ in the ear skin sections. (**A**) The integrated optical densities were measured from at least five random fields per section. (**B and C**) The integrated optical densities were measured from at least five random fields per section. (**D**) The Western blot analysis shows the protein expressions of SREBP-1, PPAR-γ, and GAPDH in the ear skins of mice in each group. GAPDH was used to confirm equal sample loading. (**E and F**) The bar graph shows the quantitative signal intensity of SREBP-1 and PPAR-γ after normalization with GAPDH. Histological examinations were performed at 400× magnification under light microscopy. The results are expressed as means ± SEM. *$p < 0.05$ compared with the NC group. †$p < 0.05$ compared with the CA group. NC: normal control; CA: C. acnes; BV1, BV10, and BV100: 1, 10, and 100 μg of bee venom mixed with Vaseline.

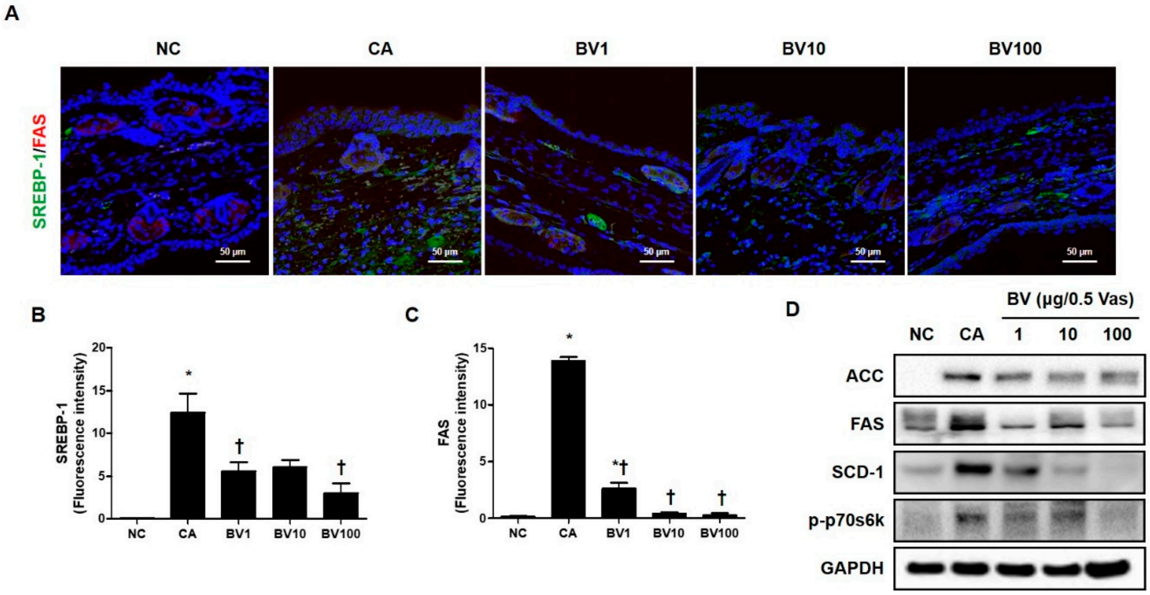

Figure S3. Effect of BV in *C. acnes*-mediated expression of lipogenesis-related genes

The inhibitory effect of BV on lipid synthesis in *C. acnes*-induced acne mice was examined by immunofluorescence. The representative immunofluorescence images show the effect of BV on the *C. acnes*-induced activation of SREBP-1 (labeled with Alexa Fluor 488, green) and FAS (labeled with Alexa Fluor 555, red). (**A-C**) The nuclei were labeled with 4,6-diamidino-2-phenylindole dihydrochloride (DAPI) (blue); Scale bar = 50 μm. (**D**) The protein level for lipid-associated factor was evaluated by the Western blot analysis. The blots were incubated with antibodies specific to ACC, FAS, SCD-1, and p70s6k. GAPDH was used as a loading control. The Results are expressed as means ± SEM. *p < 0.05 compared with the NC group. †p < 0.05 compared with the CA group. NC: normal control; CA: C. acnes; BV1, BV10, and BV100: 1, 10, and 100 μg of bee venom mixed with Vaseline.

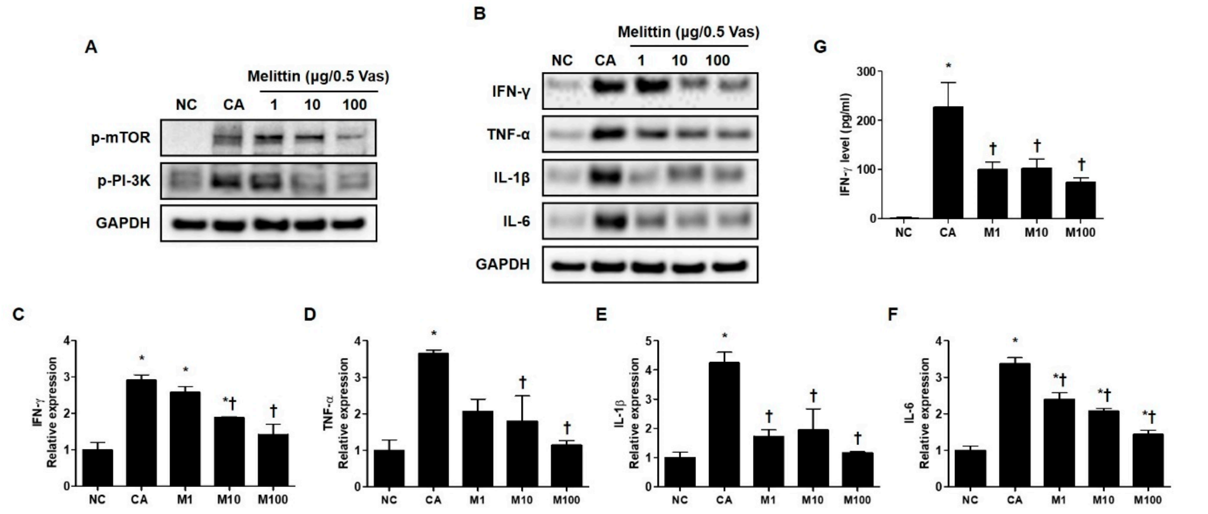

Figure S4. Inhibition of lipogenesis and inflammatory reaction by melittin in *C. acnes*-induced acne model

The mice were injected with *C. acnes*. Melittin was then applied at the indicated concentrations for 24 h. (**A**) The protein level for the lipid synthesis associated PI3K/mTOR pathway was evaluated by the Western blot. The blots were incubated with antibodies specific to PI3K and p-mTOR. GAPDH was used as a loading control. (**B**) The representative Western blot images showed that melittin attenuated the *C. acnes*-induced protein expression of IFN-γ, TNF-α, IL-1β, and IL-6 in mice. GAPDH was presented as loading control. (**C-F**) We next quantified the protein levels. (**G**) The results of ELISA demonstrate that melittin suppressed *C. acnes*-induced increased concentrations of serum- IFN-γ. The results are expressed as means ± SEM. *$p < 0.05$ compared with the NC group. †$p < 0.05$ compared with the CA group. NC: normal control; CA: *C. acnes*; M1, M10, and M100: 1, 10, and 100 μg of melittin mixed with Vaseline and applied topically to the ear surface.

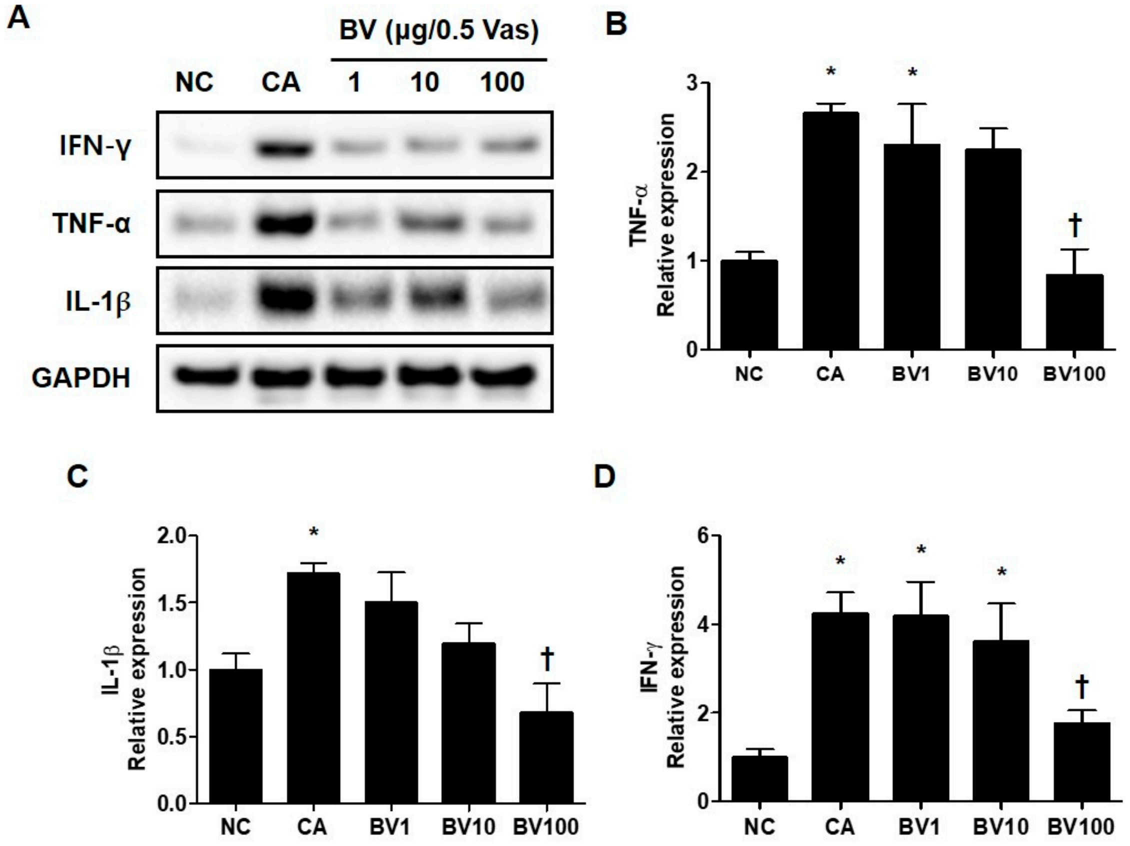

Figure S5. Inhibition of inflammatory reaction by BV in *C. acnes*-induced acne model

The mice were injected with *C. acnes*. BV was then applied at the indicated concentrations for 24 h. (**A**) The representative Western blot images showed that BV attenuated the *C. acnes*-induced protein expression of IFN-γ, TNF-α and IL-1β in mice. GAPDH was presented as loading control. (**B-D**) We next quantified the protein levels. Results are expressed as means ± SEM. *$p < 0.05$ compared with the NC group. †$p < 0.05$ compared with the CA group. NC: normal control; CA: C. acnes; BV1, BV10, and BV100: 1, 10, and 100 µg of bee venom mixed with Vaseline.

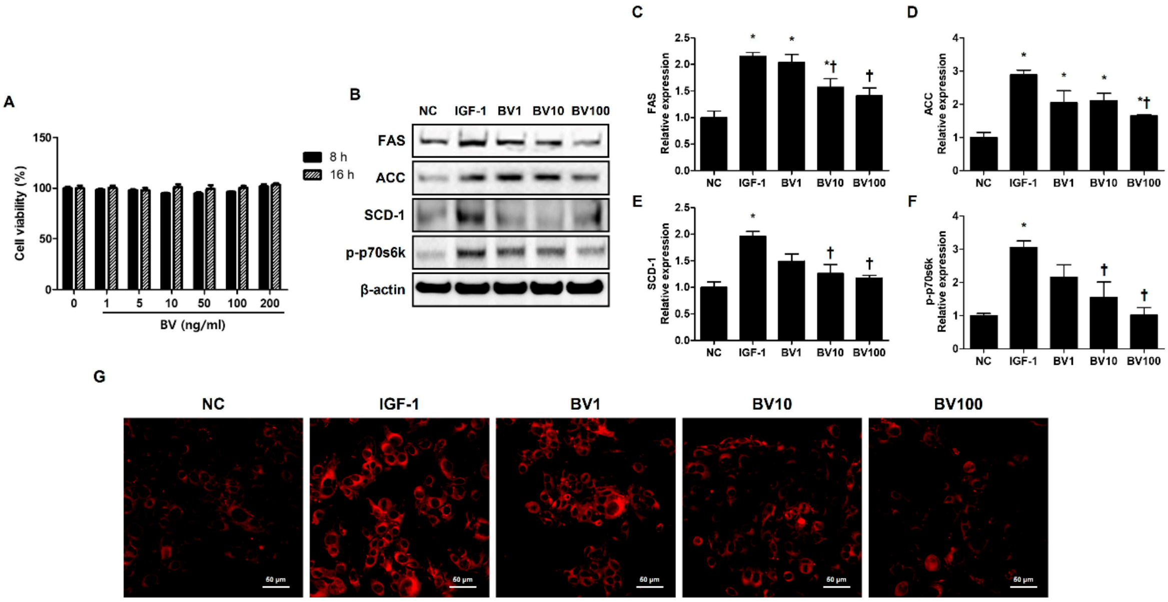

Figure S6. BV inhibits IGF-1-induced lipogenic factors in SZ95 sebocytes

(**A**) Cytotoxic effects of BV on SZ95 sebocytes. Cell viability was determined by CCK-8 assay (n = 5). (**B**) The effects of BV on the expressions of lipogenic factors, such as ACC, FAS, SCD-1, and p-p70s6k in SZ95 sebocytes that were stimulated by IGF-1. (**C-F**) We quantified the protein levels next. β-actin was presented as loading control. Intracellular lipids were detected by Nile red staining. (**G**) Representative Nile Red stain images were detected in intracellular lipids at 400× magnification. Scale bar = 50 μm. The results are expressed as means ± SEM of three independent determinations. *$p < 0.05$ compared with the NC group. NC group. †$p < 0.05$ compared with the IGF-1 group. NC: normal control; IGF-1: insulin-like growth factor-1; BV1, BV10, and BV100: 1, 10, and 100 ng·ml^{-1}.

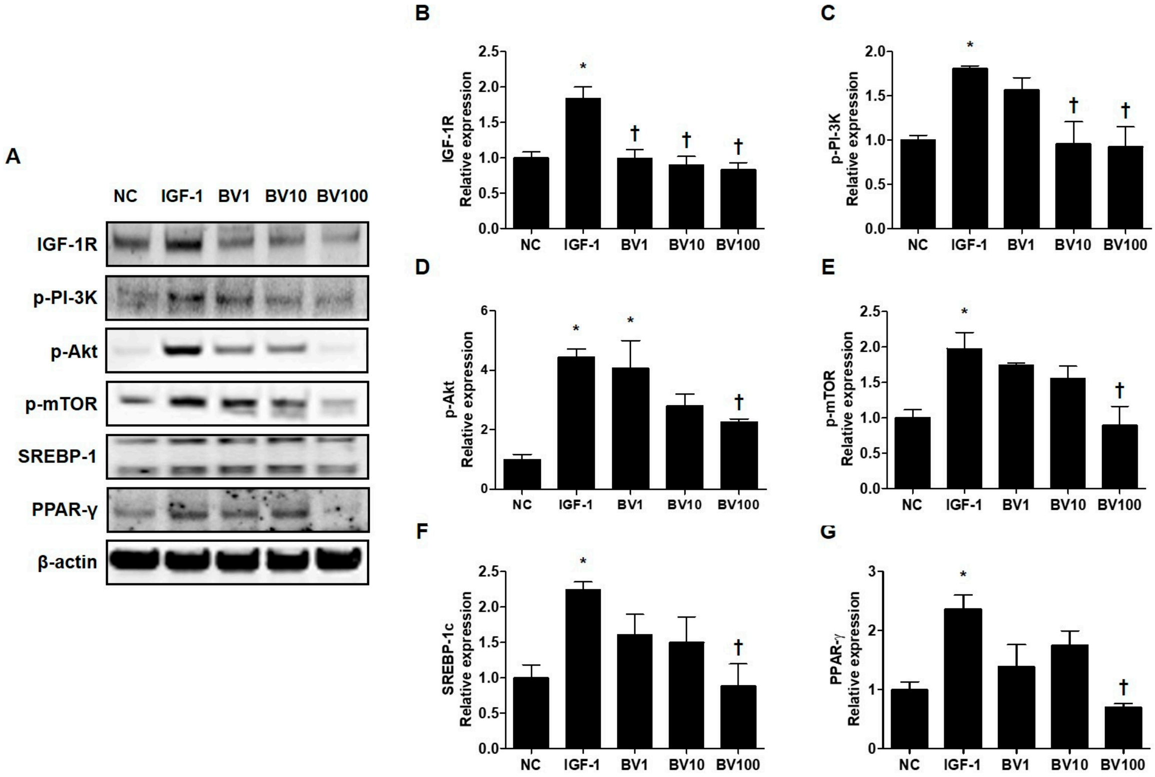

Figure S7. BV effectively inhibits the IGF-1R/Akt/mTOR/SREBP signaling pathway in IGF-1-treated SZ95 sebocytes

Western blot analysis shows that the phosphorylation of p-IGF-1R, p-PI3K, p-Akt, and p-mTOR is suppressed by treatment with BV. (**A**) Representative western blot analysis images show that treatment with BV almost completely blocked the activation of IGF-1R/Akt/mTOR/SREBP signaling pathway after treatment of SZ95 sebocytes with IGF-1. β-actin was presented as a loading control. (**B-G**) We quantified the protein levels next. The results are expressed as means ± SEM. **p* < 0.05 compared with the NC group. †*p* < 0.05 compared with the IGF-1 group. NC: normal control; IGF-1: insulin-like growth factor-1 BV1, BV10, and BV100: 1, 10, and 100 ng·ml^{-1}.

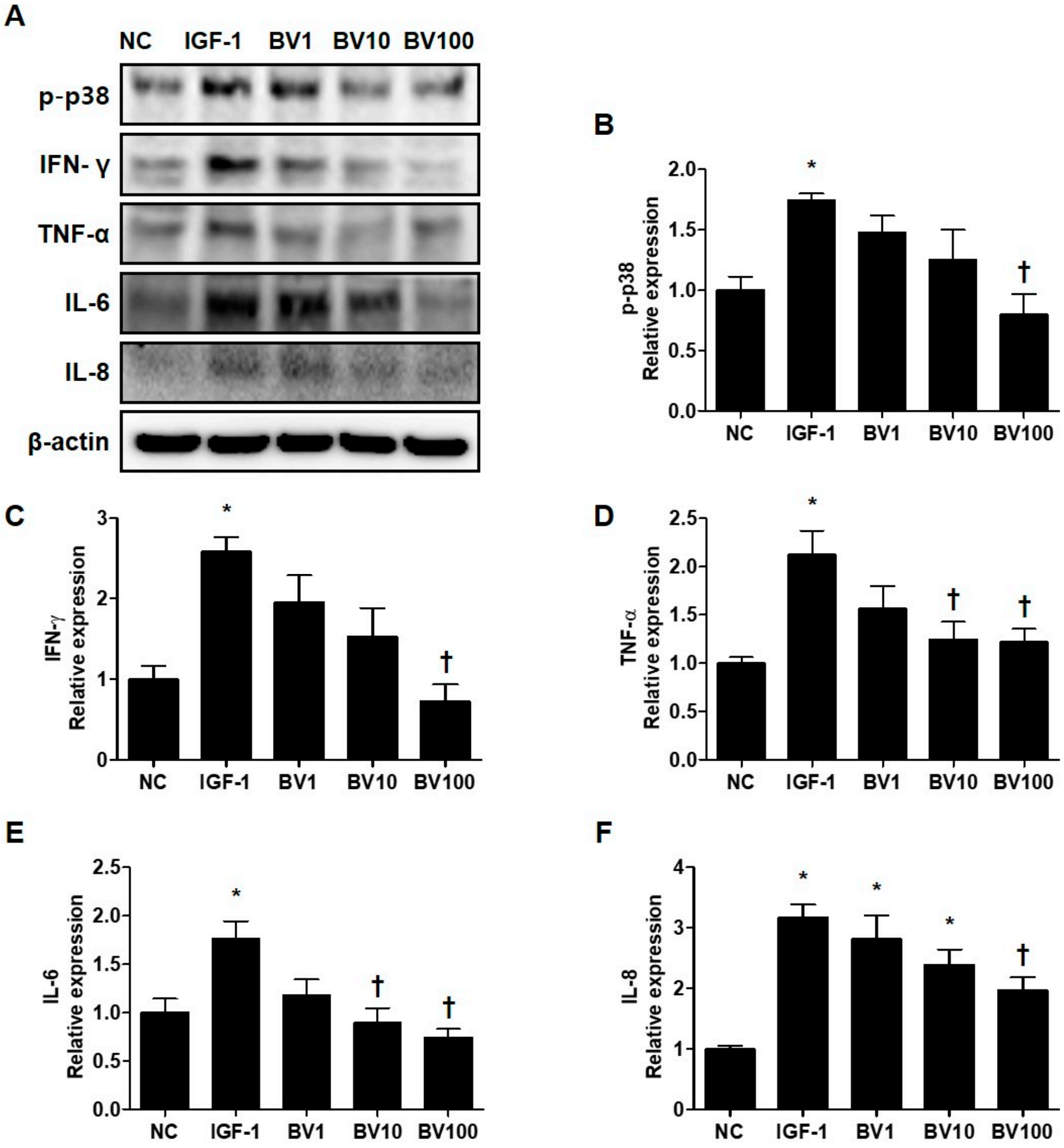

Figure S8. BV effectively inhibits the p38 MAPK and pro-inflammatory cytokines in IGF-1-treated SZ95 sebocytes

(**A**) Representative cropped Western blot analysis images show that BV inhibited the IGF-1-induced protein expressions of p38 MAPK and pro-inflammatory cytokines, such as TNF-α, IL-1β, IL-8, and IFN-γ. β-actin was presented as a loading control. (**B-F**) We quantified the protein levels next. The results are expressed as means ± SEM. *$p < 0.05$ compared with the NC group. †$p < 0.05$ compared with the IGF-1 group. NC: normal control; IGF-1: insulin-like growth factor-1; BV1, BV10, and BV100: 1, 10, and 100 $ng \cdot ml^{-1}$.
